# Ossiculoplasty for Malleus Bar with Incudostapedial Disconnection and Fused Incudomalleolar Joint

**DOI:** 10.1155/2020/8828969

**Published:** 2020-07-23

**Authors:** Yuji Kanazawa, Hitomi Matsuura, Natsumi Aiso, Masako Nakai

**Affiliations:** Department of Otolaryngology, Shiga Medical Center for Children, 5-7-30 Moriyama, Moriyama City 524-0022, Japan

## Abstract

Malleus bar is an abnormal bony connection between the malleus handle and the posterior wall of the tympanic cavity. We report a patient with a malleus bar and another malformation of the ossicles. An 11-year-old boy presented with hearing impairment since early childhood. Computed tomography (CT) revealed a malleus bar with an incudostapedial disconnection in the right ear. At tympanoplasty, the malleus bar was first identified and removed. A fused malleus-incus, not visible on the preoperative CT, was found intraoperatively. Therefore, the fused malleus-incus was removed; then, the ossicular chain was reconstructed, resulting in an improved postoperative hearing level. On preoperative CT, the disconnected incudostapedial joint had been identified, whereas the fused malleus-incus had not. Given the variations in the malleus bar anomaly of the middle ear, the surgical procedure for ossiculoplasty should be adapted intraoperatively based on any findings not visible on the preoperative CT.

## 1. Introduction

Malleus bar is a rare congenital malformation of the middle ear, consisting of a bony bar connecting the tip of the malleus handle and the posterior wall of the tympanic cavity [1]. Although congenital aural atresia or stenosis often accompanies it, intact ossicular chains have been reported with this anomaly in many cases [1–4]. In such cases, only removing the bony bar improves the hearing level [2]. However, surgical management is challenging in cases of malleus bar accompanied by other malformations of the ossicles.

We report a case of an 11-year-old boy who had malleus bar with incudostapedial disconnection and fixation of the malleus head and incus body in the right ear. Additionally, he had malleus bar with congenital aural atresia in the left ear. This patient underwent removal of the malleus bar and ossiculoplasty using a columella made of fused autoincus, resulting in improved hearing in the right ear.

## 2. Case Presentation

**Figure 1:**
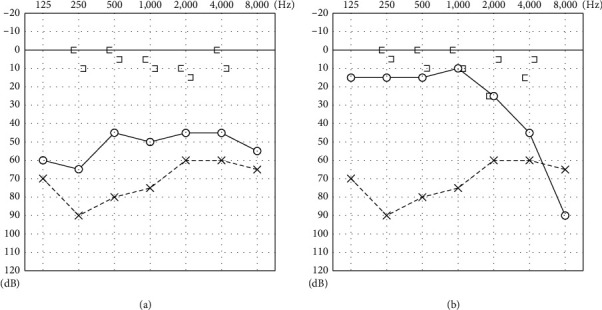
Preoperative and postoperative hearing levels: (a) preoperative pure-tone audiometry (PTA) showing bilateral conductive hearing impairment; (b) postoperative PTA showing an improvement in hearing in the right ear.

**Figure 2:**
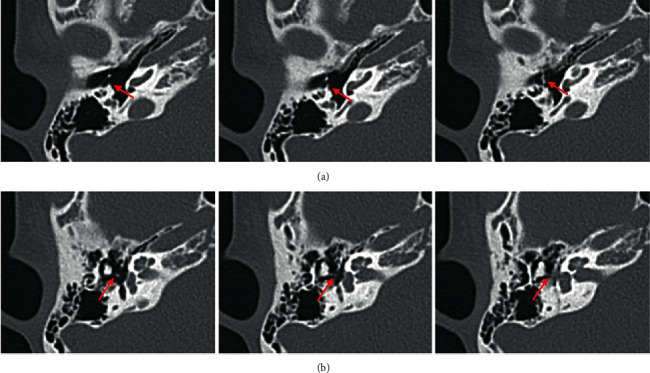
Axial view of preoperative temporal bone CT: (a) a bony bar from the posterior wall of the tympanic cavity connects to the malleus neck (arrow); (b) the long process of the incus is poorly visualized, suggesting incudostapedial disconnection (arrow).

**Figure 3:**
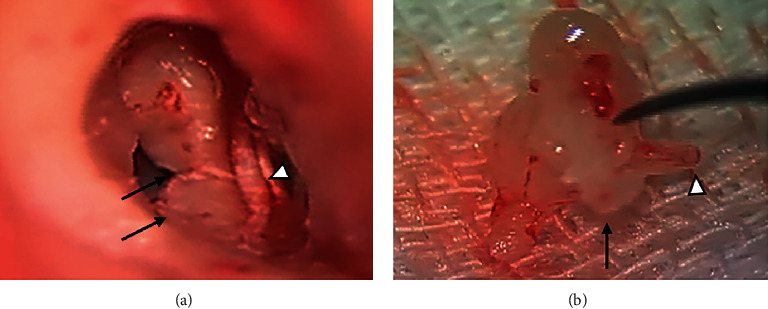
Intraoperative findings of the right middle ear: (a) the malleus neck connects to the posterior wall of the tympanic cavity via a bony bar (arrow), which is located inside the chorda tympani (arrowhead); (b) the malleus bar is shown by the arrow. The lenticular process of the incus is absent, and the long process is disconnected from the stapes (arrowhead).

An 11-year-old boy presented to our department complaining of bilateral hearing loss since early childhood. He had bilateral microtia and left congenital aural atresia. Pure-tone audiometry showed an air-bone gap of 42.5 dB in the right ear and 56.3 dB in the left ear (Figure 1). Computed tomography (CT) revealed a bony bar connecting the malleus neck to the posterior tympanic wall and a long, thin process of the incus in the right ear (Figure 2). At the right tympanoplasty (see the video in the supplemental files), we found a thick bony bar adjacent to the chorda tympani (Figure 3). The handle and neck of the malleus were disconnected, and the long process of the incus was short and connected to the stapes via fibrous tissue (Figure 3(b)). The incudomalleolar joint was missing and adhesive. After cutting the fibrous tissue connecting the incus to the stapes, the bony bar was taken down. Then, a complex of the incus and malleus, without the malleolar handle, was removed via the epitympanum after canal wall up mastoidectomy. Finally, the ossicular chain was reconstructed using a columella made of a complex of malleus and incus. Postoperatively, the air-bone gap decreased, and the hearing level was improved.

## 3. Discussion

Although there are a small number of case reports, malleus bar is rarely accompanied by other ossicular malformations. In our experience, these ossicular malformations can include disconnected incudostapedial joint, disconnected malleus neck and handle, and adhesive incudomalleolar joint. In such cases, ossiculoplasty is challenging; in addition to removal of the malleus bar, ossiculoplasty using a columella should be considered. The surgical procedure must be adapted based on an unexpected intraoperative finding, such as adhesive incudomalleolar joint, which is difficult to identify by preoperative CT.

The incudostapedial joint was missing in this case. Previous case reports have described patients with malleus bar and no other malformations of the ossicles [2, 3, 5]. In such cases, only removal of the malleus bar is needed to improve hearing. To the best of our knowledge, this is the first reported case of malleus bar with a disconnected incudostapedial joint. In such a case, reconstruction of the ossicular chain using a columella should be considered.

We found the fused incudomalleolar joint intraoperatively, making it difficult to remove the incus and malleus individually, because the incus and malleus cannot be separated at their joint. We performed not an atticotomy, but a canal wall up mastoidectomy to conserve the shape of the external auditory canal and to create a working space large enough to remove the complex. A previous case series proposed that the malleus and incus are commonly fused in cases of malleus bar with congenital aural atresia [4]. However, in cases of malleus bar without congenital aural atresia, the fused malleus-incus complex has still been reported. This finding is difficult to predict by the preoperative CT. Thus, canal wall up mastoidectomy or atticotomy may be needed when the fused malleus-incus complex is found intraoperatively.

The mechanism underlying the development of the malleus bar remains unknown; however, it is suggested to be associated with the incomplete absorption of the mesenchyme behind the malleus handle [3]. After an embryonic stage of 6.5 weeks, one part of the blastemal differentiated into a definitive cartilaginous form of the malleus and incus. Although the malleus and incus were not histologically separate at this time point, a circumferential sulcus appeared: the future incudomalleolar joint. The ossification of the malleus and incus progresses between the 4-month stage and the 6-month stage [6]. The incudomalleolar joint may be fused due to the abnormal transformation of the cartilage to bone during these fetal periods. The developmental origin of the malleus and the incus is common, except for the anterior process of the malleus, which independently develops in the membrane bone [6]. In this case, the disconnected neck and handle of the malleus could be associated with the abnormal process of establishing a connection between the anterior process and other parts of the malleus.

In conclusion, we reported a case of malleus bar with another ossicular malformation. The surgical procedure should be adapted based on intraoperative findings of other ossicular malformations unidentified by the preoperative CT. There can be variation in middle ear malformations accompanying malleus bar. Further studies are needed to investigate the varieties of malformations accompanying the malleus bar.

## Supplementary Materials

Supplementary Materials Video: ossiculoplasty in the present case.
